# Differential evolution-based optimization of corn stalks black liquor decolorization using active carbon and TiO_2_/UV

**DOI:** 10.1038/s41598-021-98006-8

**Published:** 2021-09-16

**Authors:** Mircea Teodor Nechita, Gabriel Dan Suditu, Adrian Cătălin Puițel, Elena Niculina Drăgoi

**Affiliations:** Faculty of Chemical Engineering and Environmental Protection “Cristofor Simionescu”, “Gheorghe Asachi” Technical University, Bd. Prof. Dimitrie Mangeron, No. 73, 700050 Iaşi, România

**Keywords:** Chemical engineering, Environmental chemistry, Software

## Abstract

In this work, the active carbon adsorption and TiO_2_/UV decolorization of black liquor were studied through experimental analysis (planned using Design of Experiments), modelling and optimization (with Response Surface Method and Differential Evolution). The aim is to highlight the importance of optimization methods for increasing process efficiency. For active carbon adsorption, the considered process parameters were: quantity of active carbon, dilution, and contact time. For TiO_2_ promoted photochemical decolorization the process parameters were: TiO_2_ concentration, UV path length and irradiation time. The determined models had an R squared of 93.82% for active carbon adsorption and of 92.82% for TiO_2_/UV decolorization. The optimization of active carbon resulted in an improvement from 83.08% (corresponding to 50 g/L quantity of active carbon, 30 min contact time and 200 dilution) to 100% (corresponding to multiple combinations). The optimization of TiO_2_/UV decolorization indicated an increase of efficiency from 36.63% (corresponding to 1 g/L TiO_2_ concentration, 60 min irradiation time and 5 cm UV path length) to 46.83% (corresponding to 0.4 g/L TiO_2_ concentration, 59.99 min irradiation time and 2.85 cm UV path length). These results show that the experiments and the subsequent standard RSM optimization can be further improved, leading to better performance.

## Introduction

Black liquor (BL) is one the most known by-products of the pulp and paper industry. It is highly appreciated for its caloric value as industrial fuel and correspondingly unattractive from the environmental point of view^1^. Due to their environmental toxicity, both the chemical content and the colour of the black liquor containing effluents are of major concern. Therefore, numerous physicochemical treatments and decolorization methods have been proposed over the years. Examples include: coagulation and precipitation^2,3^, electrocoagulation^4,5^, adsorption^6^, wet oxidation^7^, ozonation^8,9^, photochemical degradation^10^, biodegradation and other advanced oxidation processes^11,12^, each having its own advantages and drawbacks. In the context of technical progress of pulp and paper industry and with the continuous tightening of the environmental standards and regulations, these methods must continually prove their economical and practical viability. Thus, in order to increase efficiency and reduce costs, great efforts were made to enhance and to optimize the existing treatment methods and to find the optimal operational parameters^13–20^.

Proposed in the 60 s by Box and Hunter, one of the classical optimization approaches used for studies regarding the degradation and decolorization of pulp and paper effluents^12,21–23^ is Response Surface Method (RSM) based on central composite design (CCD)^24^. Currently, the outstanding progress of computational science and engineering allows the application of a variety of algorithms for optimizing real-world problems^25–30^. These optimizers can be classified in various ways, but the most used criterion divides then into deterministic and stochastic^31^. The difference between the two consists in the characteristics of the solutions obtained. If starting from the same point, the deterministic approaches will always provide the same solution. On the other hand, the stochastic ones will generate different solutions that can be distributed in the search space or tightly packed together. Due to their effectiveness and general applicability, the stochastic approaches are often used as alternatives to the classical optimization variants. Therefore, in this work, along RSM modelling, a stochastic approach represented by a bio-inspired metaheuristic—Differential Evolution (DE)^32^- was used as an alternative to the RSM optimization.

The goal of the current work is twofold: (i) to study and identify the optimal condition for decolorization of a black liquor obtained in laboratory from corn stalks; and (ii) to demonstrate that the classical approaches can be improved to keep up with the novel decolorization processes. To this means, a three-step procedure is used:

(i) experimental (where an experimental plan is set-up and followed). Two extensively studied decolorization procedures^10,33–36^ were selected and used in lab-scale experiments: active carbon decolorization (ACD) and TiO_2_ promoted photochemical decolorization (PCD). The role of specific key parameters and their interactions were evaluated for each procedure;
(ii) modelling (where data gathered from the previous step is statistically modelled in order to determine a set of mathematical relations that can describe the processes). The method used to perform this step is represented by RSM;
(iii) optimization (where the previously determined model in combination with an optimizer is used to identify the optimal process parameters). The algorithm used to perform the optimization is represented by DE.

The chosen decolorization methods (ACD and PCD) were selected based on their simplicity, reduced cost and ease of implementation. Moreover, the base materials for the ACD and PCD methods, active carbon and respectively TiO_2_ are highly valued, with countless applications in environmental protection. The active carbon-based materials were extensively studied as adsorbents. In order to increase their functionality, various strategies for their production and activation were developed^37,38^. In the latest years, the focus was on converting low value lignocellulosic biomass (renewable crops, agriculture waste, and invasive plants) into active carbon and on their application for wastewater treatments^39–43^. This conversion involves pyrolysis (anaerobic thermal activation or physical activation) that can be preceded or followed by complementary chemical activation^42,44^. By varying the nature of the raw materials, the pyrolysis temperature, and the nature of the chemical reagents (commonly ZnCl_2_, H_3_PO_4_, NaOH or KOH), the active carbon surface area can be controlled and enhanced^41–44^. On the other hand, TiO_2_ (titania) has multiple applications as pigment (white) and as photocatalyst^45^. Nowadays, the research is focused on new strategies to improve the photocatalytic activity^46,47^. Two main directions can be distinguished: altering the materials crystallinity (rutile/anatase ratio)^48–50^ and the use of doping with non-metals such as C, N and S, transition metals such as Al, Fe, Cu, V, Ni or noble metals^47,51–53^.

The aim of the current work is not to prove which of the two processes is better but to demonstrate that novel computational techniques can provide additional strategies to improve their efficiency beyond the standard performance levels reported so far. Since the focus is not on particular properties, the materials used (active carbon and TiO_2_) are commercially available and were used as such, without any alteration or supplementary treatment. To the author’s knowledge, the combination of ACD and PCD with the modelling/optimization techniques (RSM and DE) has never been studied in such manner before.

This work is organized as follows. "Materials and methods" section describes the materials and methods used from both an experimental and simulation point of view. "Results and discussion" section presents and discusses the results obtained from multiple perspectives: experimental, modelling and optimization. The last section concludes the paper.

## Materials and methods

### Materials

**Figure 1:**
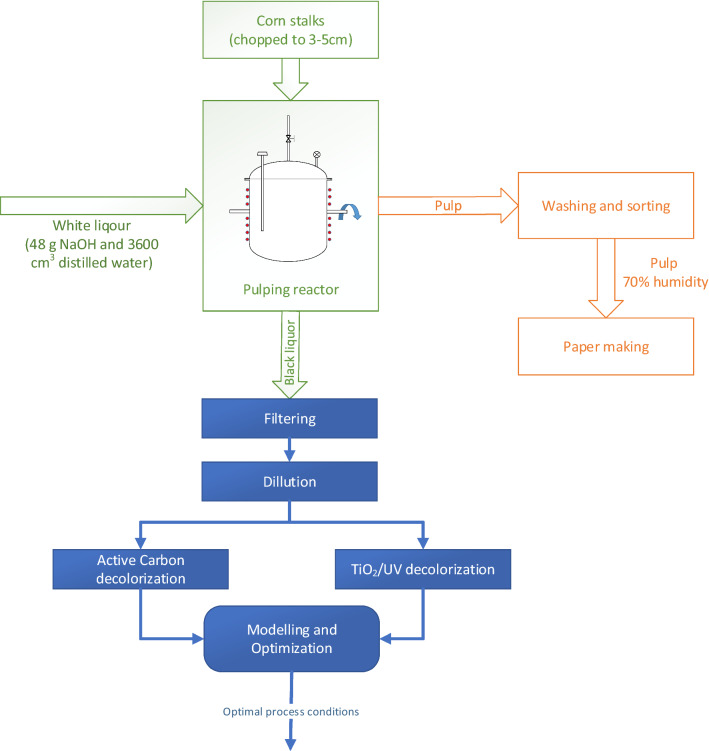
Simplified schema for processing corn stalks and black liquor.

The black liquor used in this study was obtained from a laboratory scale system for cellulose production and it was based on pulping of corn stalks. The corn stalks come from unprotected corn plantations and were gathered from the Iasi region, Romania with the permission of the farmers, following the national rules for agricultural waste collection. In a typical pulping experiment, about 440 g stalks (10% humidity) were used. The material was pulped with 48 g NaOH and 3600 cm^3^ distilled water (corresponding to 12% NaOH alkali charge and a solid to liquid ratio of 1:9). Following reactor closing, heat was applied to reach a temperature of 120 °C (25 min). This temperature was maintained for 40 min. These conditions were determined as optimum in a previous study focusing on the soda pulping of corn stalks^54^. After pulping time was completed, a sample of the liquid phase was withdrawn from the pulping reactor, cooled to room temperature and filtered to remove any remaining solids. The resulted liquor had a characteristic darkish brown colour, a relatively high alkalinity (pH = 11) a conductivity of 24.5 mS/cm and organic load (Chemical Oxygen Demand – COD = 40 g O_2_/L). The solid content of the black liquor was further determined according to the TAPPI test method (TAPPI T650, 1989). Organic to inorganic ratio was determined taking into account the ash content values (TAPPI 625 cm–85). The simplified schema of the steps used in the processing of corn stalks is presented in Fig. 1, where the blue colour indicates the focus of the current paper.

Common commercial TiO_2_ powder (M-1319) supplied at FCC purity grade by Mayam^55^ was used as such. The powder was characterized by SEM and EDX analysis and, in a previous study, was successfully applied for photochemical decolorization of methylene blue^56^.

The experiments were performed using irregular shape particles of active carbon supplied by Buzău Romcarbon Company (Romania), active carbon that was characterized in the study of^57^: specific microporous volume 0.48 cm^3^/g, total microporous volume 0.66 cm^3^/g, mean pore size 1.62 nm, BET surface 1403 m^2^/g, external surface 38 m^2^/g and total surface 631 m^2^/g. Prior to the experimental study, the particles were classified by sieving, the average diameter ranging between 2.5 and 3.15 mm.

### Equipment

The UV light source was a Biocomp UV-lamp with a wavelength of 253.7 ± 0.8 nm. An analogue UV light sensor GUVA S12SD was used to measure the intensity of incident UV radiation. The UV–VIS spectra and the absorbance values were recorded using a JASCO V-550 UV–VIS spectrophotometer. The COD mg O_2_/L was measured using a standard Hach-Lange kit LCK 114. A BHG Hermle Z 229 Centrifuge: 220 V, 50 Hz, 1.1 A, 240 W, maximum number of revolutions 15,000 rpm was also used.

### Experimental design

In order to determine the optimal parameters for BL decolorization three representative independent variables were considered for each method, as depicted in Table 1. These parameters and their limits were selected based the data provided by literature^58^. Following the design of experiments approach (DOE) proposed by Box and Hunter^24^, a minimum number of experiments were statistically programmed as presented in Table 2, where η_ACD_ (%) represents the decolorization efficiency for the active carbon decolorization and η_PCD_ (%) for the TiO_2_ promoted photochemical decolorization. In Table 2 both the coded and the decoded variables were presented, the notations being the same as the ones used in Table 1. The bold columns from Table 2 indicate the experimental results obtained with the variables determined by the DOE approach.

**Table 1:** Designated variables and their variation range for the chosen decolorization methods.

Independent variables	Measure units	Notation	Range		Symbol
			from	to	
**Active carbon decolorization (ACD)**					
Active carbon concentration	g/L	[AC]_x1_	5	50	AC
Dilution	ratio	[Ct]_x2_	1:100	1:200	Dil
Contact time	min	[Dil]_x3_	10	30	Ct
**TiO**_**2**_**promoted photochemical decolorization (PCD)**					
TiO_2_ concentration	g/L	[TiO_2_]_x1_	1	2	TiO_2_
UV path length	cm	[hUV]_x2_	5	25	hUV
Irradiation time	min	[It]_x3_	15	60	It

**Table 2:** Experiment planning and results.

No	Coded variable			ACD				PCD			
				Decoded variables			η_ACD_ (%)	Decoded variables			η_PCD_ (%)
	x_1_	x_2_	x_3_	[AC]_x1_	[Ct]_x2_	[Dil]_x3_		[TiO_2_]_x1_	[It]_x2_	[hUV]_x3_	
1	1	1	1	50	30	200	**83.08**	2	60	25	**19.83**
2	− 1	1	1	5	30	200	**57.40**	1	60	25	**20.62**
3	1	− 1	1	50	10	200	**69.74**	2	15	25	**16.88**
4	− 1	− 1	1	5	10	200	**33.54**	1	15	25	**18.41**
5	1	1	− 1	50	30	100	**81.22**	2	60	5	**33.90**
6	− 1	1	− 1	5	30	100	**52.27**	1	60	5	**36.63**
7	1	− 1	− 1	50	10	100	**60.68**	2	15	5	**18.50**
8	− 1	− 1	− 1	5	10	100	**21.48**	1	15	5	**20.45**
9	α	0	0	54.8	20	150	**80.53**	2.11	37.5	15	**19.39**
10	− α	0	0	0.176	20	150	**13.99**	0.89	37.5	15	**24.90**
11	0	α	0	27.5	32.15	150	**78.58**	1.5	64.8	15	**19.52**
12	0	− α	0	27.5	7.85	150	**65.70**	1.5	10.15	15	**17.23**
13	0	0	α	27.5	20	210.75	**72.65**	1.5	37.5	27.15	**18.61**
14	0	0	− α	27.5	20	89.25	**74.24**	1.5	37.5	2.85	**35.01**
15	0	0	0	27.5	20	150	**68.88**	1.5	37.5	15	**18.74**
16	0	0	0	27.5	20	150	**69.28**	1.5	37.5	15	**19.52**

The bold indicated in the section Experimental design.

### Experimental procedure

The BL was used as such, underprivileged of any pH chemical regulations, at room temperature. Bi-distilled water was used to reach the required dilution ratio for each experiment. In order to avoid settling (and to ensure a constant exposure of the mixture) the slurry was stirred constantly during all the experiments involving the presence of TiO_2_ powder or active carbon particles.

In the case of active carbon decolorization, for well-defined periods of time, 100 mL samples of specifically diluted BL solutions were mixed with the adequate amount of active carbon, according to the data presented in Table 2. Disposable disc filters 0.45 µm were used for particles separation.

In the case of TiO_2_ promoted photochemical decolorization, 50 mL samples of BL solutions (with 1:100 dilution ratio) were mixed with the adequate amount of TiO_2_ and placed below the UV source for the corresponding period of time. After irradiation, the TiO_2_ powder was separated from the solutions using a centrifugal separator. The required UV path length that gives the intensity of the incident UV radiation was attained by changing the distance between the UV source and the sample under study.

### Chemical assays

**Figure 2:**
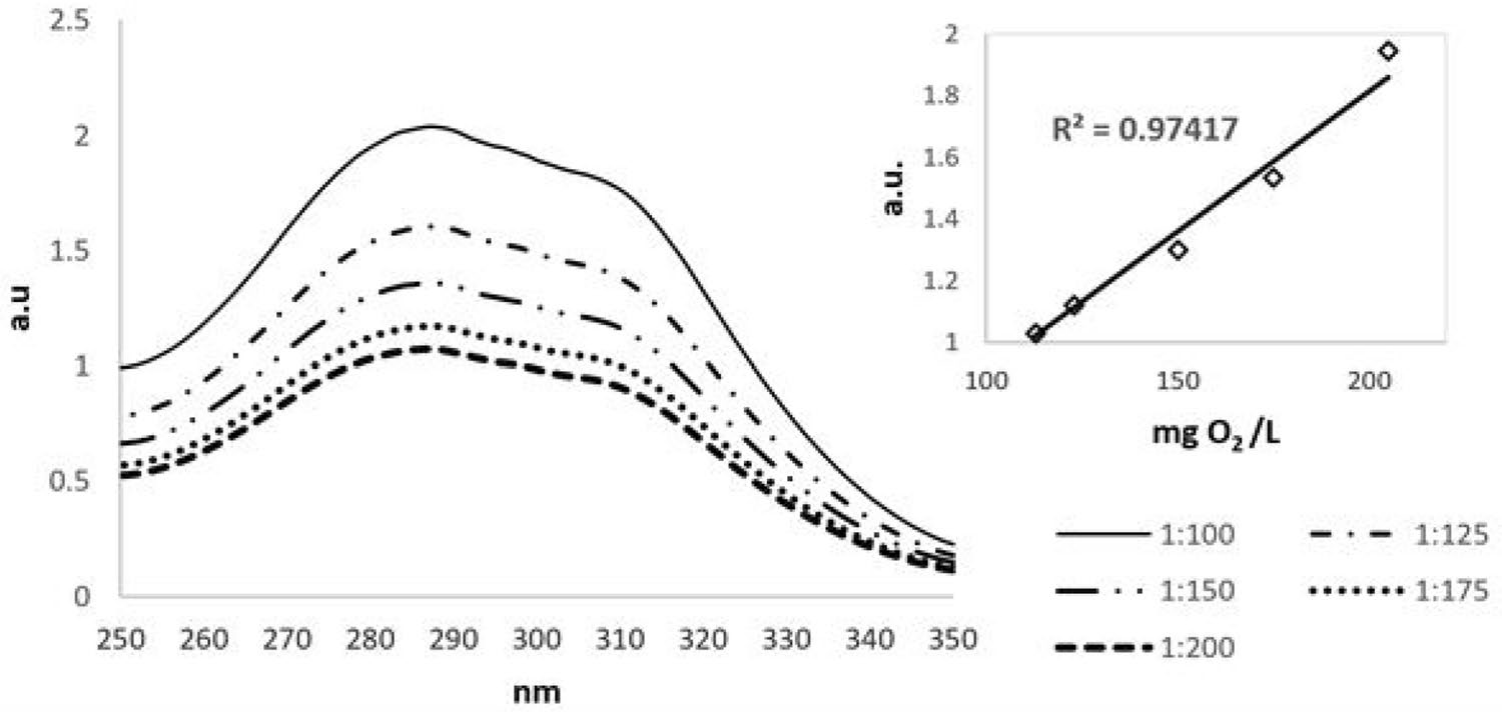
UV–VIS spectra of BL at different dilution ratio. The inset displays the correlation between COD and the absorbance at the corresponding dilution.

The BL decolorization was checked by measuring the absorbance of the solution given by the lignin content at 280 nm (UV_280_) (Fig. 2). The correlation between COD and absorbance was determined at different dilution ratios in order to establish a calibration curve that validates the accuracy of decolorization efficiency calculations. The coefficient of determination (R^2^) was 0.97, in accordance with the literature reported values^21^. The efficacy of BL decolorization was calculated using the following equation:

1 $${{\eta (\% )}} = \frac{{\left[ {{\text{UV}}_{280} } \right]_{0} - \left[ {{\text{UV}}_{280} } \right]}}{{\left[ {{\text{UV}}_{{{280}}} } \right]_{0} }} \cdot 100$$

where $\left[ {{\text{UV}}_{280} } \right]_{0}$ and $\left[ {{\text{UV}}_{280} } \right]$ are the absorbance’s recorded before and after each experiment.

### Software and algorithm

The MINITAB package (Minitab Institute, USA) was chosen to implement the response surface method algorithm. In addition, the process was optimized with a second method represented by DE, an efficient metaheuristic approach, that was successfully used (simple or in combination with other approaches) for optimization and modelling of a wide range of systems: robot control^59^, water quality monitoring^60^, adsorption processes^61^. Examples of DE application in chemical engineering can be found in^62^. The DE based software used was developed in^63^ in combination with artificial neural networks (ANNs) and applied for predicting the liquid crystalline property of some organic compounds. Distinctively, in this work, the ANN is replaced by the model determined with MINITAB package and the DE variant (SADE) performs only the process optimization part.

DE is inspired from the Darwinian principle of evolution^32^ and it works with a population of potential solutions that it is evolved (through a series of steps that include mutation, crossover and selection) until a stop criterion is reached. In the first step, the potential solutions (which will be further referred as individuals) are initialized using a random based procedure. This population then undergoes a mutation procedure. DE has many mutation variants and, in this work, two differential terms combined with a randomly selected based vector was used. This combination is also known as the rand/2 version. Equation (2) describes the mutation equation used.

2 $$\omega_{i} = \alpha + F \cdot \left( {\beta + \gamma } \right)$$

where *α* is the base vector, *F* is the scaling factor (one of the control parameters of DE), and *β*, *γ* are the differential terms. The differential term is created by subtracting a randomly selected vector with another one.

After that, the features of the mutated and current individuals are combined to create a new population called trial. This is the crossover step and the variant used in this work is the binomial crossover.

In the next step, the trial and the current population undergo a one-to-one comparison where the best individuals are selected to form the next generation. The measure used to determine the best individuals is represented by the fitness function. For the current work, the fitness function represents the output of the regression model generated by Minitab software.

**Figure 3:**
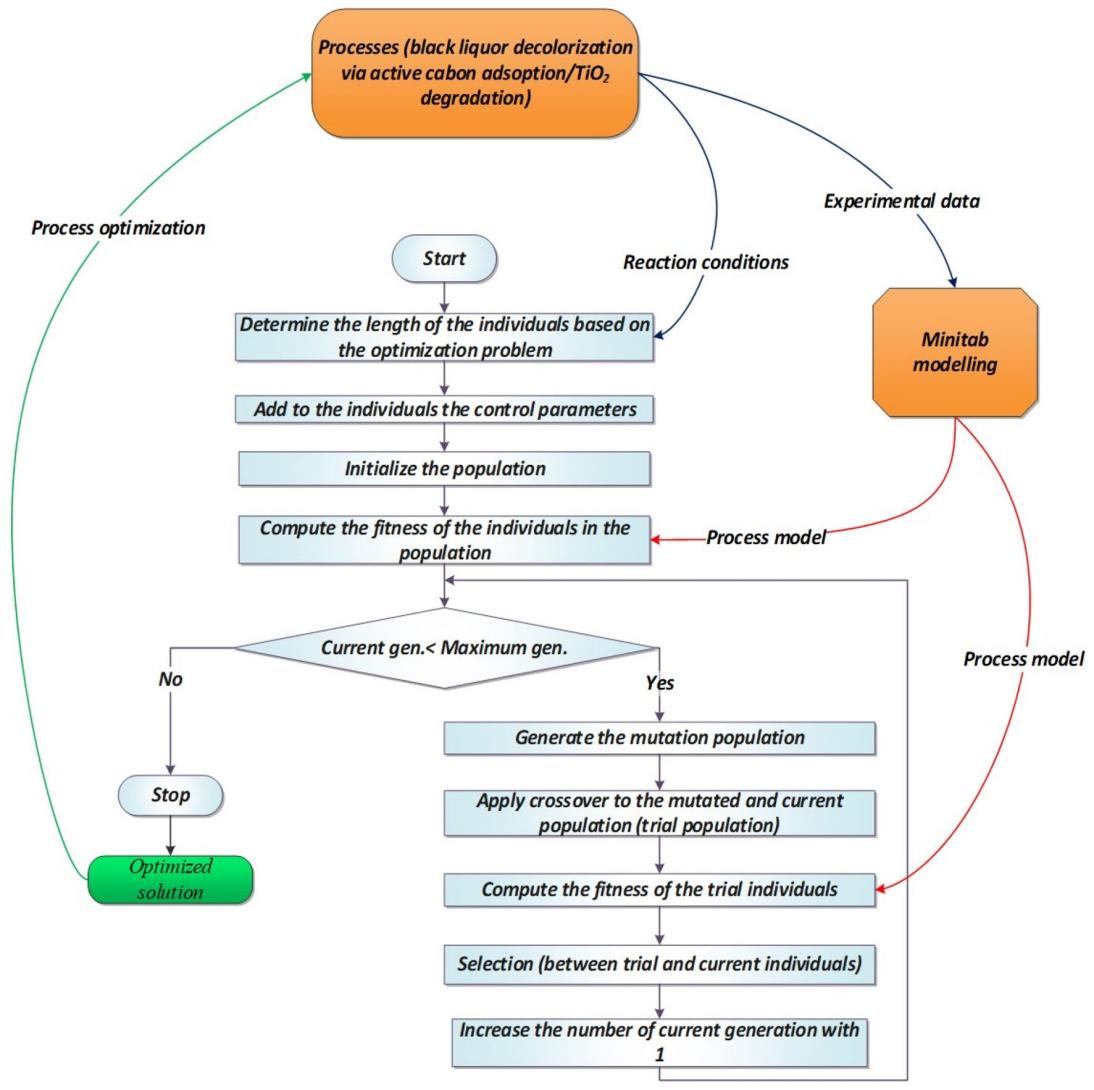
Scheme of the overall optimization procedure.

One of the main characteristics of the SADE version is represented by the use of self-adaptability to determine the values of the control parameters. In this manner, the difficult task of manually setting the optimal values for the control parameters is automatized. A simplified schema of approach used in this work is presented in Fig. 3.

## Results and discussion

### ACD vs PCD

Evidently, the basic principles of the BL decolorization methods selected for this study makes a comparison attempt to be rather impractical. Furthermore, the chosen parameters and their range of variation make the straightforward comparison between ACD and PCD quite difficult.

However, from the experimental data obtained on the samples with the dilution ratio (1:100) used for both methods (the experiments from 5 to 8 in Table 2) the superiority of ACD is clearly established. The results are comparable in one case only, for the experimental data set no. 8, when the values for the ACD and PCD parameters were set for minimum values. Evidently, in terms of decolorization efficiency, the ACD method exhibits better performances (83.08% vs. 36.63%).

When it comes to PCD, it should be mentioned that, commonly, the method involves the use of additional chemical oxidants: Fenton’s reagent, hydrogen peroxide and other combinations that favours the formation of OH radicals and other short-lived radical species, which highly elevates the methods efficacy. However, no additional chemicals (reagents, pH regulators) were used during this study, since our goal was not necessary to compare two well-known methods but to use and apply classic and modern optimization techniques in order to find their optimal parameters. Therefore, no claim that one method is better than the other will conclude our work.

### Response surface method

A full second-order polynomial model was obtained by multiple regression technique for three parameters using the MINITAB package. For the ACD method, the regression equation in terms of actual factors (uncoded units) is presented below:

3 $$\begin{aligned} \eta_{{{\text{ACD}}}} \left( \% \right) = & 0.{2} + {2}.{833} \cdot {\text{AC}} + {1}.{59} \cdot {\text{Ct}} - 0.{124} \cdot {\text{Dil}} - 0.0{3}0{73} \cdot {\text{AC}}^{{2}} + 0.0{13}0 \cdot {\text{Ct}}^{{2}} \\ & \quad + 0.000{87} \cdot {\text{Dil}}^{{2}} - 0.0{143} \cdot {\text{AC}} \cdot {\text{Ct}} - 0.000{14} \cdot {\text{AC}} \cdot {\text{Dil}} - 0.00{478} \cdot {\text{Ct}} \cdot {\text{Dil}} \\ \end{aligned}$$

**Figure 4:**
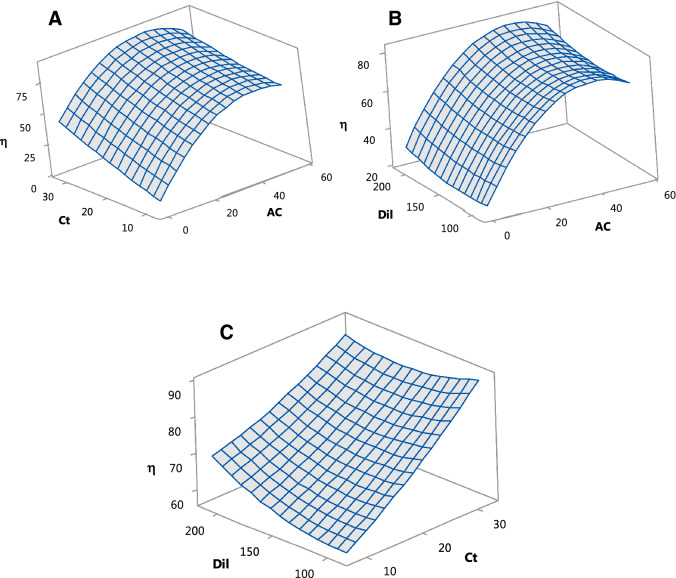
(**A**) Surface plot efficiency vs Ct and AC, at Dil = 1:150; (**B**) Surface plot efficiency vs Dil and AC, at Ct = 20 min; (**C**) Surface plot efficiency vs Dil and Ct, at AC = 27.5 g/L.

By setting one parameter at a constant value equal to the median value of the interval of variation, three dimensional plots were drawn (Fig. 4A,B,C). This allows the visualization of maximum and/or minimum points that leads to accurate identification of the optimal values and shows the influence of the selected parameters on the BL decolorization efficiency.

The response surface plots showing the evolution of the decolorization efficiency as a function of contact time and active carbon concentration at constant dilution ration 1:150 is displayed in Fig. 4A. As expected, the increase of Ct and AC have a substantial influence on efficiency, its maximum value (over 75%) being reached after 30 min at 40 g/L adsorbent concentration. Figure 4B shows the influence of dilution ratio and active carbon concentration on decolorization after 20 min of contact time. The maximum value of decolorization efficiency was reached at 1:200 Dil in presence of 40 g/L active carbon. When AC was held constant at 27.5 g/L the efficiency reached nearly 95% after 30 min and 1:200 Dil as presented in Fig. 4C.

The data presented in Table 3 represent the best five sets of Minitab optimization results. For all the optimization data provided in each case, two solutions were experimentally validated in order to confirm the results.

**Table 3:** The RSM optimization for active carbon decolorization of BL.

Solution no	AC, g/L	Ct, min	Dil, ratio	η_ACD,_ (%)-predicted	η_ACD_, (%)-experimental
1	38.43	32.15	89.25	92.24	92.3
2	38.12	32.15	210.75	89.70	91.1
3	47.53	32.15	89.25	89.68	-
4	37.32	32.15	210.75	89.68	-
5	29.77	31.95	89.65	89.59	-

**Figure 5:**
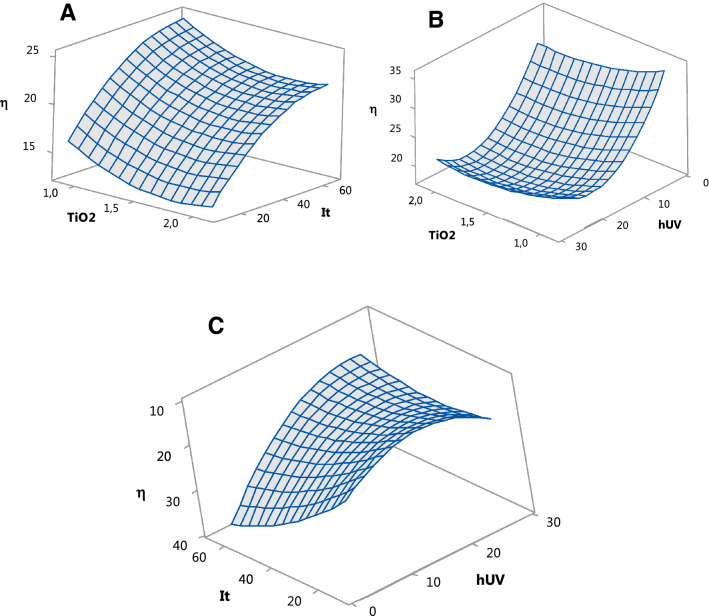
(**A**) Surface plot efficiency vs It and TiO_2_, at hUV = 15 cm; (**B**) Surface plot efficiency vs hUV and TiO_2_, at It = 37.5 min; (**C**) Surface plot efficiency vs It and hUV, at TiO_2_ = 1.5 g/L.

For the PCD method, the regression equations in terms of actual factors (uncoded units) is presented in Eq. (4). Figures 5A, 5B and 5C display the variation of BL decolorization efficiency for PCD as a function of two variables.

4 $$\begin{aligned} \eta_{{{\text{PCD}}}} \left( \% \right) = & {33}.0 - {16}.{3} \cdot {\text{TiO}}_{{2}} + 0.{6}00 \cdot {\text{It}} - {1}.{299} \cdot {\text{hUV}} + {4}.{3}0 \cdot {\text{TiO}}_{{2}}^{{2}} \\ & \quad - 0.00{292} \cdot {\text{It}}^{{2}} + 0.0{424} \cdot {\text{hUV}}^{{2}} - 0.000 \cdot {\text{TiO}}_{{2}} \cdot {\text{It}} \\ & \quad + 0.0{59} \cdot {\text{TiO}}_{{2}} \cdot {\text{hUV}} - 0.0{1468} \cdot {\text{It}} \cdot {\text{hUV}} \\ \end{aligned}$$

Figure 5A shows the response surface plots for decolorization efficiency as a function of irradiation time and TiO_2_ concentration at a constant UV path length of 15 cm. It can be noticed that the efficiency rises with the increase of It and it is higher at lower values of TiO_2_. The surface plot in Fig. 5B shows the influence of TiO_2_ concentration and UV path length after 37.5 min of irradiation. Once more, the efficiency is higher at lower values of TiO_2_ and tends to increase with the decrease of hUV. When TiO_2_ is held constant at 1.5 g/L (Fig. 5C), the efficiency grows with the increase of irradiation time and with the decrease of the UV path length.

The best five sets of results for the Minitab optimization are presented in Table 4.

**Table 4:** The RSM optimization results for PCD of BL.

Solution no	TiO_2_, g/L	It, min	hUV, cm	η_PCD,_ (%)-predicted	η_PCD_, (%)-experimental
1	0.89	64.84	2.85	42.57	43.2
2	2.10	64.84	2.85	38.64	38.8
3	0.89	45.46	2.85	37.99	-
4	0.89	36.39	27.15	22.71	-
5	0.89	27.59	27.15	22.59	-

### Differential evolution

In order to determine the optimal configuration of parameters leading to the maximization η_PCD_, (%) for both PCD and ACD approaches, DE in combination with the regression equations generated by Minitab (Eqs. 3, 4) was applied. The control parameters values were automatically adjusted by the software using a self-adaptive procedure^63^. The settings used for DE optimization were: number of individuals in the population = 30, number of iterations = 50. These values were selected based on the author’s expertise and practical aspects. The parameters included into the optimization process are the same as the process parameters considered in the modelling phase: AC, Ct, Dil (for ACD) and TiO_2_, It, hUV (for PCD).

For both PCD and ACD, the DE based optimization procedure was applied in three cases: (i) the limits of the operating conditions were the same as in the experimental data (Case 1); (ii) the limits were extended to ± 20% (extrapolation) (Case 2); and (iii) the quantity of active reagents added was limited (1–20 g/L activated carbon for ACD and 0.4–1 g/L TiO_2_ for PCD) (Case 3). Tables 5 and 6 list five solutions obtained in each of these three cases. It is worth mentioning that, due to the stochastic nature of the DE base algorithm, at each run, different solutions can be obtained. Therefore, DE is not limited by a pre-specified number of solutions and can provide various configurations that lead to very similar results.

**Table 5:** DE optimization results for ACD of BL.

Case	Solution no	AC, g/L	Ct, Min	Dil, ratio	η_ACD,_ (%)-predicted	η_ACD_, (%)-experimental
Case 1	1	19.07	47.51	169.59	100	99.03
	2	36.3	41	138.16	100	-
	3	25.87	42.6	131.24	100	-
	4	31.27	39.15	104.08	99.98	-
	5	7.29	52.42	121.7	99.97	98.87
Case 2	1	56.76	50.48	134.08	100	97.42
	2	32.21	42.92	191.68	100	-
	3	25.94	43.69	152.67	100	-
	4	19.46	46.83	157.38	99.97	-
	5	55.7	48.1	120.17	99.95	98.24
Case 3	1	4.84	55.18	135.77	100	98.76
	2	8.98	54.14	172.91	100	-
	3	12.34	50.02	137.35	100	-
	4	17.99	46.49	135.48	100	-
	5	19.99	47.38	183.31	99.98	98.69

**Table 6:** DE optimization results for PCD of BL.

Case	Solution no	TiO_2_, g/L	It, Min	hUV, cm	η_PCD,_ (%)-predicted	η_PCD_, (%)-experimental
Case 1	1	0.89	59.99	2.85	41.64	40.84
	2	0.90	58.83	3.03	40.98	-
	3	0.90	57.74	2.96	40.88	-
	4	0.99	59.82	3.04	40.45	-
	5	1.10	57.74	3.33	38.70	36.42
Case 2	1	0.71	71.98	2.28	46.58	45.98
	2	0.72	68.42	2.30	45.88	-
	3	0.75	70.08	2.34	45.77	-
	4	0.78	69.11	2.46	45.08	-
	5	0.87	69.03	3.41	42.34	42.4
Case 3	1	0.40	59.99	2.85	46.83	46.22
	2	0.49	57.28	2.88	45.09	-
	3	0.48	55.41	3.57	43.53	-
	4	0.43	48.59	3.11	43.26	-
	5	0.56	54.91	3.65	42.35	41.75

As it can be observed from the experimental validation, there is an acceptable error between the predictions and the actual values. In addition, compared with the solutions provided by the RSM approach, DE is able to find optimal values in a wide range of combinations. For example, for the ACD process, in Case 1, the process efficiency varies between 99.97 and 100%, while the values for the identified parameters varies between [7.29, 31.27] for AC, [41, 52.42] for Ct and [104.08, 169.59] for Dil. This implies that the efficiency function is multimodal and that that there are multiple combinations for the parameters values that lead to the same result. Taking into consideration the economic aspect, the process optimization can be transformed from a single-objective (maximum efficiency) to a multi-objective problem (highest efficiency with the minimum of resources consumed).

A literature analysis regarding similar strategies for decolorization using active carbon (Table 7) and TiO_2_ (Table 8) indicate that the obtained results are similar with other studies. However, the BL decolorization efficiency is strongly influenced by: (i) the black liquor source and/or the preparation methods; (ii) the source and preparation methods used for the materials (active carbon or TiO_2_); (iii) the scheme used for decolorization treatment (e.g., UV-Fenton-TiO_2_, free TiO_2_/UV, enzyme-AC).

**Table 7:** Comparison of various black liqueurs decolorization by adsorption on active carbons.

Author, ref. no	Black liquor source	Active carbon source/system	Decolorization* (%)
Shivayogimath and Navaneet^64^	Paper mill effluent, India	Laboratory prepared activated carbon, from teakwood sawdust	83.19
Singh^65^	Paper mill effluent, India	Laboratory prepared activated carbon, from coconut jute	90.1
Zhang and Chuang^34^	Soft wood Kraft pulp mill, China	Commercial, Aldrich	95
Gupta et al.^66^	Kraft process-based pulp and paper mill, India	Laboratory prepared activated carbon, from plastic mix waste	96.48
Sari et al.^67^	Plant reactor for bioethanol production, Indonesia	Laboratory prepared activated carbon, from black liquor coagulated sludge, immobilized enzymes	97.7
This study	Laboratory produced from corn stalks, Romania	Commercial, Romcarbon Company	99.03
Mehmood et al.^68^	Paper and board mill, Pakistan	Combined physicochemical treatment, granular AC	99.5

*The highest reported value.

**Table 8:** Comparison of photochemical decolorization of various black liqueurs.

Author, ref. no	Black liquor source	TiO_2_ source/system	Decolorization/degradation* (%)
Maulidiyah et al.^69^	Laboratory produced from oil palm empty fruit bunches, Indonesia	FeO.TiO_2_, natural mineral	31.579
This study	Laboratory produced from corn stalks, Romania	Free TiO_2_, Mayam	46.22
Ksibi et al.^36^	Pulp and paper firm, Tunisia	Free TiO_2_, Degussa P25	56
Peralta-Zamora et al.^58^	Pulp and paper industry, Sao Paulo, Brazil	Free TiO_2_, Degussa P25	75
Arutanti et al.^70^	Pilot plant reactor, second-generation bioethanol production, Indonesia	Laboratory prepared TiO_2_ nanoparticles, Fenton system	90
Chang et al.^33^	Laboratory prepared synthetic lignin wastewater (Aldrich)	Free TiO_2_, Degussa P25	99

*The highest reported value.

As it can be observed from Table 7, in case of activated carbon, the efficiency obtained in this work is comparable with more complex strategies that include immobilized enzymes and combined physiochemical treatment. On the other hand, for TiO_2_, the obtained degradation efficiency is lower compared with the works where TiO_2_, Degussa was used. However, compared with TiO_2_ Degussa, the TiO_2_ used in this work is approximately 40 times cheaper.

## Conclusions

In this work, the active carbon decolorization and TiO_2_ promoted photochemical decolorization of black liquor obtained from laboratory pulping of corn stalks was studied using experimental combined with Design of Experiments, Response Surface Methodology and Differential Evolution algorithm. The optimization simulations for both processes were experimentally validated, the obtained errors being in an acceptable interval (< 5%). Compared with RSM, the DE based approach is more flexible (allows a wide range for parameter limits) and it is better at exploring the search space, being able to determine multiple combinations of solutions leading to similar outputs. Moreover, for active carbon, an improvement from 81.27% to 100% and for TiO_2_/UV decolorization from 36.63% to 46.83% was obtained, proving that the application of state-of-the-art computational approaches can lead to significant improvements and that they can be efficiently used raise performance of various decolorization processes. The mechanisms and kinetics of active carbon absorption and of TiO_2_/UV photochemical degradation and/or mineralization (considering the optimal conditions identified in this study) represents the subject of a future work that will include a detailed HPLC analysis of black liquor before and after decolorization.

## Data Availability

All the data this article is based on is presented in the text, tables and figures.

## Code availability

https://elenadragoi.ro/CV/Documents/AITB-%20ANN_DE.7z.

## References

[1] Zaied M, Bellakhal N (2009). Electrocoagulation treatment of black liquor from paper industry. J. Hazard. Mater..

[2] Ganjidoust H, Tatsumi K, Yamagishi T, Gholian RN (1997). Effect of synthetic and natural coagulant on lignin removal from pulp and paper wastewater. Water Sci. Technol..

[3] Garg A, Mishra IM, Chand S (2010). Effectiveness of coagulation and acid precipitation processes for the pre-treatment of diluted black liquor. J. Hazard. Mater..

[4] Azadi Aghdam M, Kariminia H-R, Safari S (2016). Removal of lignin, COD, and color from pulp and paper wastewater using electrocoagulation. Desalin. Water Treat..

[5] Shankar R, Singh L, Mondal P, Chand S (2014). Removal of COD, TOC, and color from pulp and paper industry wastewater through electrocoagulation. Desalin. Water Treat..

[6] Mohan SV, Karthikeyan J (1997). Removal of lignin and tannin colour from aqueous solution by adsorption onto activated charcoal. Environ. Pollut..

[7] Garg A, Mishra IM, Chand S (2007). Catalytic wet oxidation of the pretreated synthetic pulp and paper mill effluent under moderate conditions. Chemosphere.

[8] Kreetachat T (2007). Effects of ozonation process on lignin-derived compounds in pulp and paper mill effluents. J. Hazard. Mater..

[9] De Santos R. W, Poznyak T, Chairez I, Córdova RI (2009). Remediation of lignin and its derivatives from pulp and paper industry wastewater by the combination of chemical precipitation and ozonation. J. Hazardous Mater..

[10] Kansal SK, Singh M, Sud D (2008). Studies on TiO_2_/ZnO photocatalysed degradation of lignin. J. Hazard. Mater..

[11] Merayo N, Hermosilla D, Blanco L, Cortijo L, Blanco Á (2013). Assessing the application of advanced oxidation processes, and their combination with biological treatment, to effluents from pulp and paper industry. J. Hazard. Mater..

[12] Baycan PN, Akten D (2011). Optimization of TiO_2_/Fe(III)/solar UV conditions for the removal of organic contaminants in pulp mill effluents. Desalination.

[13] Darvishmotevalli M, Zarei A, Moradnia M, Noorisepehr M, Mohammadi H (2019). Optimization of saline wastewater treatment using electrochemical oxidation process: prediction by RSM method. MethodsX.

[14] Ghasemi S (2020). Design, operation, performance evaluation and mathematical optimization of a vermifiltration pilot plan for domestic wastewater treatment. J. Environ. Chem. Eng..

[15] Kloch M, Toczyłowska-Mamińska R (2020). Toward optimization of wood industry wastewater treatment in microbial fuel cells—mixed wastewaters approach. Energies.

[16] Panepinto, D. *et al.* in *Frontiers in Water-Energy-Nexus—Nature-Based Solutions, Advanced Technologies and Best Practices for Environmental Sustainability* 231–233 (Springer, 2020).

[17] Shomar B, Al-Darwish K, Vincent A (2020). Optimization of wastewater treatment processes using molecular bacteriology. J. Water Process Eng..

[18] Wang J (2020). Multivariate optimization of the pulse electrochemical oxidation for treating recalcitrant dye wastewater. Sep. Purif. Technol..

[19] Zhang M-H, Dong H, Zhao L, Wang D-X, Meng D (2019). A review on Fenton process for organic wastewater treatment based on optimization perspective. Sci. Total Environ..

[20] Zhou X, Hou Z, Lv L, Song J, Yin Z (2020). Electro-Fenton with peroxi-coagulation as a feasible pre-treatment for high-strength refractory coke plant wastewater: Parameters optimization, removal behavior and kinetics analysis. Chemosphere.

[21] Torrades F, Saiz S, García-Hortal JA (2011). Using central composite experimental design to optimize the degradation of black liquor by Fenton reagent. Desalination.

[22] Kim S-C (2016). Application of response surface method as an experimental design to optimize coagulation–flocculation process for pre-treating paper wastewater. J. Ind. Eng. Chem..

[23] Subramonian W, Wu TY, Chai S-P (2017). Photocatalytic degradation of industrial pulp and paper mill effluent using synthesized magnetic Fe_2_O_3_-TiO_2_: Treatment efficiency and characterizations of reused photocatalyst. J. Environ. Manage..

[24] Box GEP, Hunter JS (1957). Multi-Factor Experimental Designs for Exploring Response Surfaces. Ann. Math. Stat..

[25] Curteanu, S., Dragoi, E.-N., Blaga, A. C., Galaction, A. I. & Cascaval, D. in *Artificial Neural Networks* (ed Hugh Cartwright) 115–138 (Springer US, 2021).

[26] Godini K, Azarian G, Kimiaei A, Dragoi EN, Curteanu S (2021). Modeling of a real industrial wastewater treatment plant based on aerated lagoon using a neuro-evolutive technique. Process Saf. Environ. Prot..

[27] Li L, Rong S, Wang R, Yu S (2021). Recent advances in artificial intelligence and machine learning for nonlinear relationship analysis and process control in drinking water treatment: a review. Chem. Eng. J..

[28] Malviya A, Jaspal D (2021). Artificial intelligence as an upcoming technology in wastewater treatment: a comprehensive review. Environ. Technol. Rev..

[29] Thon C, Finke B, Kwade A, Schilde C (2021). Artificial intelligence in process engineering. Adv. Intell. Syst..

[30] Butnariu C, Lisa C, Leon F, Curteanu S (2013). Prediction of liquid-crystalline property using support vector machine classification. J. Chemom..

[31] Mirjalili S (2017). Salp Swarm Algorithm: a bio-inspired optimizer for engineering design problems. Adv. Eng. Soft.

[32] Storn, R. & Price, K. Differential evolution-a simple and efficient adaptive scheme for global optimization over continuous spaces. (Berkley, 1995).

[33] Chang C-N (2004). Decolorizing of lignin wastewater using the photochemical UV/TiO_2_ process. Chemosphere.

[34] Zhang Q, Chuang KT (2001). Adsorption of organic pollutants from effluents of a Kraft pulp mill on activated carbon and polymer resin. Adv. Environ. Res..

[35] Ugurlu M, Gurses A, Yalcin M, Dogar C (2005). Removal of phenolic and lignin compounds from bleached Kraft Mill effluent by fly ash and sepiolite. Adsorption.

[36] Ksibi M (2003). Photodegradation of lignin from black liquor using a UV/TiO2 system. J. Photochem. Photobiol. A.

[37] Marsh, H. & Rodriguez-Reinoso, F. *Activated Carbon*. (Elsevier, 2006).

[38] Cecen, F. & Aktas, O. *Activated Carbon for Water and Wastewater Treatment. Integration of Adsorption and Biological Treatment*. (Wiley, 2012).

[39] Osman AI, Farrell C, Al-Muhtaseb AAH, Harrison J, Rooney DW (2020). The production and application of carbon nanomaterials from high alkali silicate herbaceous biomass. Sci. Rep..

[40] Nguyen TTH (2021). Converting biomass of agrowastes and invasive plant into alternative materials for water remediation. Biomass Convers. Biorefinery.

[41] Nizam NUM, Hanafiah MM, Mahmoudi E, Halim AA, Mohammad AW (2021). The removal of anionic and cationic dyes from an aqueous solution using biomass-based activated carbon. Sci. Rep..

[42] Gale M, Nguyen T, Moreno M, Gilliard-AbdulAziz KL (2021). Physiochemical properties of biochar and activated carbon from biomass residue: influence of process conditions to adsorbent properties. ACS Omega.

[43] Osman AI (2020). Upcycling brewer's spent grain waste into activated carbon and carbon nanotubes for energy and other applications via two-stage activation. J. Chem. Technol. Biotechnol..

[44] Osman AI (2019). Production and characterisation of activated carbon and carbon nanotubes from potato peel waste and their application in heavy metal removal. Environ. Sci. Pollut. Res..

[45] Tan, L.-L., Wong, V. L. & Phang, S. J. in *Handbook of Nanotechnology Applications* (eds Woei Jye Lau, Kajornsak Faungnawakij, Kuakoon Piyachomkwan, & Uracha Rungsardthong Ruktanonchai) 25–65 (Elsevier, 2021).

[46] Nemiwal M, Zhang TC, Kumar D (2021). Recent progress in g-C3N4, TiO2 and ZnO based photocatalysts for dye degradation: strategies to improve photocatalytic activity. Sci. Total Environ..

[47] Zoubi WA, Al-Hamdani AAS, Sunghun B, Ko YG (2021). A review on TiO_2_-based composites for superior photocatalytic activity. Rev. Inorganic Chem..

[48] Hwang JY (2021). Crystal phase-dependent generation of mobile OH radicals on TiO2: revisiting the photocatalytic oxidation mechanism of anatase and rutile. Appl. Catal. B.

[49] Yaemsunthorn K, Kobielusz M, Macyk W (2021). TiO_2_ with tunable anatase-to-rutile nanoparticles ratios: how does the photoactivity depend on the phase composition and the nature of photocatalytic reaction?. ACS Appl. Nano Mater..

[50] Lei Y (2021). Controllable one-step synthesis of mixed-phase TiO2 nanocrystals with equivalent anatase/rutile ratio for enhanced photocatalytic performance. Nanomaterials.

[51] Piątkowska A, Janus M, Szymański K, Mozia S (2021). C-, N- and S-doped TiO2 photocatalysts: a review. Catalysts.

[52] Osman AI, Skillen NC, Robertson PKJ, Rooney DW, Morgan K (2020). Exploring the photocatalytic hydrogen production potential of titania doped with alumina derived from foil waste. Int. J. Hydrogen Energy.

[53] Salomatina EV (2021). Preparation and photocatalytic properties of titanium dioxide modified with gold or silver nanoparticles. J. Environ. Chem. Eng..

[54] Chesca A-M, Nicu R, Tofanica BM, Puitel AC, Gavrilescu D (2018). Optimization of soda pulping process of corn staks by response surface modelling. Cellul. Chem. Technol..

[55] Mayam. *Mayam organic & pure cosmetic ingredients.* http://www.mayam.eu (2021).

[56] Atomi AI, Suditu GD, Puiţel AC, Nechita MT (2018). Experimental study on TiO2 promoted photo-degradation of methylene blue. Bull. Romanian Chem. Eng. Soc..

[57] Secula, M. S., Cagnon, B., Cretescu, I., Diaconu, M. & Petrescu, S. Removal of an acid dye from aqueous solutions by adsorption on a commercial granular activated carbon: equilibrium, kinetic and thermodynamic study. *Sci. Study Res. Chem. Chem. Eng. Biotechnol. Food Ind.* **12**, 307 (2011).

[58] Peralta-Zamora P (1998). Evaluation of ZnO, TiO2 and supported ZnO on the photoassisted remediation of black liquor, cellulose and textile mill effluents. Chemosphere.

[59] Neri F, Mininno E (2010). Memetic compact differential evolution for cartesian robot control. IEEE Comput. Intell. Mag..

[60] Yazdi J (2018). Water quality monitoring network design for urban drainage systems, an entropy method. Urban Water J..

[61] Bleotu I, Dragoi EN, Mureşeanu M, Dorneanu S-A (2018). Removal of Cu(II) ions from aqueous solutions by an ion-exchange process: modeling and optimization. Environ. Prog. Sustain. Energy.

[62] Dragoi EN, Curteanu S (2016). The use of differential evolution algorithm for solving chemical engineering problems. Rev. Chem. Eng..

[63] Drăgoi E-N, Curteanu S, Lisa C (2012). A neuro-evolutive technique applied for predicting the liquid crystalline property of some organic compounds. Eng. Optim..

[64] Shivayogimath, C. & Bhandari, N. B. Adsorption studies of paper mill effluent on teakwood sawdust activated carbon. *Int. J. Appl. Sci. Eng. Res.* **3**, 994–1004 (2014).

[65] Singh TS (2006). Investigations on reduction of colour from pulp and paper mill effluent by activated coconut jute carbon. J. Water Supply Res. Technol. AQUA.

[66] Gupta V, Bhardwaj NK, Rawal RK (2021). Removal of colour and lignin from paper mill wastewater using activated carbon from plastic mix waste. Int. J. Environ. Sci. Technol..

[67] Sari, A. A., Hanifah, U., Parmawati, Y. & Permadi, R. in *Key Engineering Materials.* 402–407 (Trans Tech Publ).

[68] Mehmood K (2019). Treatment of pulp and paper industrial effluent using physicochemical process for recycling. Water.

[69] Maulidiyah, M., Mardhan, F., Natsir, M., Wibowo, D. & Nurdin, M. in *Journal of Physics: Conference Series.* 012017 (IOP Publishing).

[70] Arutanti O (2020). Advanced degradation of lignin from palm oil mill effluent (POME) by a combination of photocatalytic-fenton treatment and TiO2 nanoparticle as the catalyst. Water Air Soil Pollut..

